# Supplementary material for: Graphene quantum dot electrochemiluminescence increase by bio-generated H2O2 and its application in direct biosensing
Source: R Soc Open Sci. 2020 Jan 29;7(1):191404. doi: 10.1098/rsos.191404 (PMC7029901; doi:10.1098/rsos.191404)
Supplement: Figure S1 A) CVs of the electrolysis of 1.0 mg mL−1 GOQD at a scan rate of 50 mV s−1; B) CVs of Ti foil after GOQD electrolysis in 0.067 M pH~7 PBS.;Figure S2 FTIR spectra of GQD and GOQD.;Figure S3 Raman spectra of GQD and GOQD.;Figure S4 XPS spectra of (A) GOQD and (B) GQD.;Figure S5 Photoluminesc [file rsos191404supp1.doc]

**Supplementary Materials**

**Graphene quantum dot electrochemiluminescence increase by bio-generated H_2_O_2_ and its application in direct biosensing**

Shanli Yang^a^, Mingfu Chu^a^, Jie Du^a^, Yingru Li^a^, Tao Gai^a^, Xinxin Tan^a^, Binyuan Xia^a^,*, Shaofei Wang^a^,*

^a^Institute of Materials, China Academy of Engineering Physics, Jiangyou 621907, Sichuan, P. R. China

————————————

^*^ Corresponding author.

Binyuan Xia, E-mail: ybinxia@caep.cn

Shaofei Wang, E-mail: wangshaofei@caep.cn

Graphene oxide quantum dot (GOQD) was prepared according to Liu’s previous work as follows (Liu et al., 2013): after 1 g of a graphite nanoparticle (GNP) was added to a solution of H_2_SO_4_ (34 mL) and NaNO_3_ (0.75 g) in a 250 mL flask, the mixture solution was put in an ice-water bath and vigorously stirred using a magnetic stirring bar. KMnO_4_ (5 g) was added slowly, and then the temperature then increased to 35 ° C. After the mixture was stirred for 2 h, DI water (50 mL) was added to the ice bath. This was followed by the dropwise addition of 30 wt% H_2_O_2_ (4 mL) to the above solution. The mixture was transferred to a 1:10 HCl/water solution (500 mL) and then washed with DI water under centrifugation at 20000 rpm until the pH of the suspensions reached 7. Following 3 h of sonication, the suspension was centrifuged at 10000 rpm for 30 min to collect a stable GOQD solution from the supernatant.

The cyclic voltammogram (CV) of GOQD electrolysis on Ti foil has been shown in Figure S1A. The peak currents persistent decrease with successive potential sweeps, which indicates the persistent deposition of some poor conductive material directly onto Ti foil; moreover, one anodic peak (1) and two cathodic peaks (2 and 3) can be observed. To confirm the deposition material and the redox peaks, the Ti foil after GOQD electrolysis is then scanned in 0.067M pH 7 phosphate buffer solution. As shown in Figure S1B, the pair of reversible redox peaks (1 and 2) still exist while the irreversible cathodic peak 3 (~ −1.0 V) disappears, thus the cathodic current peak 3 can be attributed to the irreversible electroreduction of GOQD to GQD, and the anodic peak 1 and cathodic peak 2 are ascribed to the redox pair of some oxygen-containing groups (e.g., phenolic hydroxyl groups ) on the GQD which are too stable to be reduced by the cyclic voltammetry, similar to the previous report on the electroreduction of graphene oxide to graphene (Liu et al., 2013). And it is reasonable to speculate that the electrodeposition material on Ti foil is GQD.

To further confirm our speculation, the original GOQD and the obtained GQD have been characterized by FTIR, Raman, XPS and PL.

The FTIR spectra of GOQD and GQD are shown in Figure S2. Compared with GQD, the predominant adsorptions of oxygen-containing groups on GOQD, such as 3200−3800 cm^−1^ (O-H stretching), 1750 cm^−1^ (C=O stretching), 1490 cm^−1^ (O-H deformation), and 1101 cm^−1^ (C-O stretching) have been largely decreased, confirming again the reduction of GOQD to GQD by removing most of the oxygen-containing groups on GOQD through the electroreduction process.

Raman method can evaluate the out-of-order degree of carbon atom through the intensity ratio (I_D_/I_G_) of D band (sp^3^ carbon atom stretching) to G band (sp^2^ carbon atom stretching) (Dresselhaus et al., 2013). As shown in Figure S3, GOQD and GQD exhibits typical D band and G band adsorptions at around 1290 cm^−1^ and 1600 cm^−1^; however, the I_D_/I_G_ decreases significantly from 1.22 of GOQD to 1.01 of GQD for the formation of more sp^2^ domains in GQD, further confirming the electroreduction of GOQD to GQD.

The XPS of (A) GOQD and (B) GQD are shown in Figure S4. The C 1s XPS of GOQD and GQD demonstrate the presence of four types of carbon bonds: C-C/C=C, C-O, C=O and O-C=O. However, the bands associated with oxygenated groups in the spectrum of GQD are much weaker than those in the spectrum of GOQD, indicating again the removal of the oxygen-containing groups during the electroreduction process.

Figure S5 shows the PL spectra of GOQD/Ti foil, GQD/Ti foil and the supernatant after centrifugation of the GQD/Ti foil. When 275 nm is set as the excited wavelength, GOQD has a wide and low PL emission at around 445 nm; however, due to the disappearance of large amounts of electron-withdrawing groups (e.g., COOH and C=O group) and the increase of conjugated effect on GQD, the electron cloud density on the whole conjugated system rises, thus the excitation energy of GQD decreases, resulting in evident PL enhancement of GQD. Moreover, to test the stability of GQD on Ti foil, PL spectrum of the supernatant after centrifugation of the GQD/Ti foil also has been shown in Figure S5. No luminescence from the supernatant can be observed, suggesting the robust adhesion of GQDs to Ti foil.

**Reference**

Liu, F., Jang, M.H., Ha, H.D., Kim, J.H., Cho, Y.H., Seo, T.S., 2013. Adv. Mater. 25 3657-3662.

Liu, C.B., Wang, K., Luo, S.L., Tang, Y.H., Chen, L.Y., 2011. Small 7 1203-1206.

Dresselhaus, M.S., Jorio, A., Hofmann, M., Dresselhaus, G., Saito, R., 2010. Nano Letters 10 751-758.

**Figure S1 A) CVs of the electrolysis of 1.0 mg mL^−1^ GOQD at a scan rate of 50 mV s^−1^; B) CVs of Ti foil after GOQD electrolysis in 0.067 M pH~7 PBS.**

**Figure S2 FTIR spectra of GQD and GOQD.**

**Figure S3 Raman spectra of GQD and GOQD.**

**Figure S4 XPS spectra of (A) GOQD and (B) GQD.**

**Figure S5 Photoluminescence spectra of GOQD/Ti foil, GQD/Ti foil and the supernatant after centrifugation of the GQD/Ti foil.**

**Figure S6 (A) CVs of GQD and GQD/GOx on Ti foil in N_2_-saturated 0.067 M pH~7 PBS; (B) CVs of GQD/GOx on Ti foil at different scan rates: 0.05 V s^-1^, 0.1 V s^-1^, 0.2 V s^-1^, 0.3 V s^-1^, 0.4 V s^-1^ and 0.5 V s^-1^ respectively.**
